# Effects of Freshwater Clam Extract Supplementation on Time to Exhaustion, Muscle Damage, Pro/Anti-Inflammatory Cytokines, and Liver Injury in Rats after Exhaustive Exercise

**DOI:** 10.3390/molecules18043825

**Published:** 2013-03-26

**Authors:** Kuo-Chin Huang, Wen-Tien Wu, Fwu-Lin Yang, Yi-Han Chiu, Tai-Chu Peng, Bang-Gee Hsu, Kuang-Wen Liao, Ru-Ping Lee

**Affiliations:** 1 Department of Biological Science and Technology, National Chiao Tung University, Hsinchu 30068, Taiwan; 2 Holistic Education Center, Mackay Medical College, New Taipei City 25245, Taiwan; 3 Department of Orthopedics, Buddhist Tzu Chi General Hospital, Hualien 97004, Taiwan; 4 Intensive Care Unit, Buddhist Tzu Chi General Hospital, Taipei Branch, Taipei 23142, Taiwan; 5 Department of Nursing, St. Mary’s Medicine, Nursing and Management College, Yi-Lan 26644, Taiwan; 6 Department of Nursing, Tzu Chi University, Hualien 97004, Taiwan; 7 Department of Nephrology, Buddhist Tzu Chi General Hospital, Hualien 97004, Taiwan

**Keywords:** *Corbicula fluminea*, anti-inflammatory, endurance performance, blood glucose

## Abstract

The potent anti-inflammatory activities and tissue-protective effects of freshwater clams (*Corbicula fluminea*) have been well reported. The aim of this study was to determine the effects of freshwater clam extract (FCE) supplementation on time to exhaustion, muscle damage, pro- and anti-inflammatory cytokines, and liver injury in rats after exhaustive exercise. Thirty-two rats were divided into four groups: sedentary control (SC); SC group with FCE supplementation (SC+FCE); exhaustive exercise (E); and E group with FCE supplementation (E+FCE). The SC+FCE and E+FCE groups were treated with gavage administration of 20 mg/kg for seven consecutive days. Blood samples were collected for the evaluation of biochemical parameters. The cytokine levels of TNF-α and IL-10 were also examined. Twenty-four hours after exhaustive exercise, the rat livers were removed for H & E staining. The FCE supplementation could extend the time to exhaustion in exercised rats. The levels of CPK, LDH, AST, ALT, lactate, TNF-α and H & E stains of the liver injury were significantly decreased in the E+FCE group, but the blood glucose and IL-10 were significantly higher in comparison with the E group. This study suggests that FCE supplementation may improve endurance performance and reduce exercise-induced muscle damage, inflammatory stress and liver injury.

## 1. Introduction

Freshwater clams (*Corbicula fluminea*) are a widely consumed shellfish in Asia. A number of *in vitro* and *in vivo* studies have found that freshwater clams possess many medical and biological effects, including cholesterol-lowering [1], hepatoprotective [2], and anti-tumorigenic properties [3]. Current evidence demonstrates that freshwater clams have potent anti-inflammatory activities, which was illustrated in a variety of inflammation model systems. A previous study reported that freshwater clam extract (FCE) decreased the thiobarbituric acid reactive substances (TBARS) and excessive inflammation in CCl_4_-induced hepatitis in rats [2]. Besides, Peng *et al*. [4] indicated that FCE decreased ischemia reperfusion injury in liver cells and suppressed the release of the pro-inflammatory TNF-α cytokine while increasing the concentration of the anti-inflammatory IL-10 cytokine. Additionally, the administration of 20 mg/kg freshwater clam extract could be used to reduce the plasma TNF-α and decrease the levels of AST, ALT and LDH in conscious rats after hemorrhagic shock [5].

The beneficial effects of regular physical exercise have been well recommended for reducing the risk of cardiovascular diseases, diabetes mellitus and cancer [6]. Nevertheless, an acute bout of exhaustive exercise is known to provoke transient inflammation *in vivo* [7], muscle damage [8], delayed onset muscle soreness (DOMS) [9], and even liver injury in rats [10]. Many studies have demonstrated that muscle damage and DOMS can result in loss of muscle force and significant pain [11,12]. Muscle soreness and damage probably represent considerable obstacles to exercise performance. Currently, the role of inflammatory related cytokines, such as IL-6, TNF-α, IL-1β, and IL-10, in muscle damage after heavy exertion is well documented [13]. Hamada *et al*. [14] and Cannon *et al.* [15] suggested that pro-inflammatory cytokines are elevated immediately after intense exercise and may play a role in initiating the breakdown of damaged muscle tissue. It was also reported that long-term endurance exercise may induce muscular injury [8,16]. Muscle damage with the production of inflammatory mediators in exhaustive exercise may lead to increased pain and performance deficits in muscle function [17]. Presently there are multiple proposed methods for treating muscle damage and exercise-induced inflammation, including cryotherapy [18], anti-inflammatory medication [19], hyperbaric oxygen [20], and ultrasound [21]. However, effective treatment has not been well established.

Nowadays, nutrients as ergogenic aids provide athletes and their scientific consultants with competitive advantages [22]. Various nutritional products have been used to assess their capability to attenuate the indicators of physiologic stress while supporting exercise performance [23]. However, because many of these products do not appear to greatly improve important hematological and inflammatory markers, muscle fatigue, or physical performance, the efficacy of ergogenic aids is still in question. Freshwater clam extract, which has garnered a great deal of attention as a food-derived agent with positive health benefits, has been intensively studied [1,4,5,24]. In the interest of the protective and anti-inflammatory effects of FCE administration were further investigated in this experiment. Therefore, the aim of this study was to evaluate the effects of FCE supplementation on endurance performance, muscle damage, inflammatory cytokines and the liver injury in rats following acute exercise.

## 2. Results and Discussion

### 2.1. Effects of FCE on Body Weight

The initial body weights for the rats in the SC, SC+FCE, E, and E+FCE groups were 281.67 ± 4.01, 287.50 ± 5.74, 284.17 ± 5.07, and 286.67 ± 2.47 grams, respectively. After seven consecutive days of 20 mg/kg/day FCE administration, the body weight gains in the SC+FCE and E+FCE groups were significantly increased compared to the control group (*p* < 0.05, Table 1). However, the body weight gain was not significantly different between the E and SC groups (*p* > 0.05, Table 1).

**Table 1:** Comparative body weight changes in different groups of rats.

	Sedentary Control			Exhaustive Exercise	
	SC (*n* = 8)	SC+FCE (*n* =8)		E (*n* = 8)	E+FCE (*n* = 8)
Initial Body Weight (g)	281.67 ± 4.01	287.50 ± 5.74		284.17 ± 5.07	286.67 ± 2.47
Final Body Weight (g)	285.00 ± 4.47	293.36 ± 6.79		287.50 ± 4.96	295.00 ± 2.24
Weight Gain (g)	3.33 ± 1.05	5.83 ± 1.54		3.33 ± 1.05	8.33 ± 1.05 ^a,b^

Each value represents means ± SEM. ^a^ *p* < 0 .05 *vs.* group SC. ^b^ *p* < 0.05 *vs.* group E.

### 2.2. Time to Exhaustion

**Figure 1:**
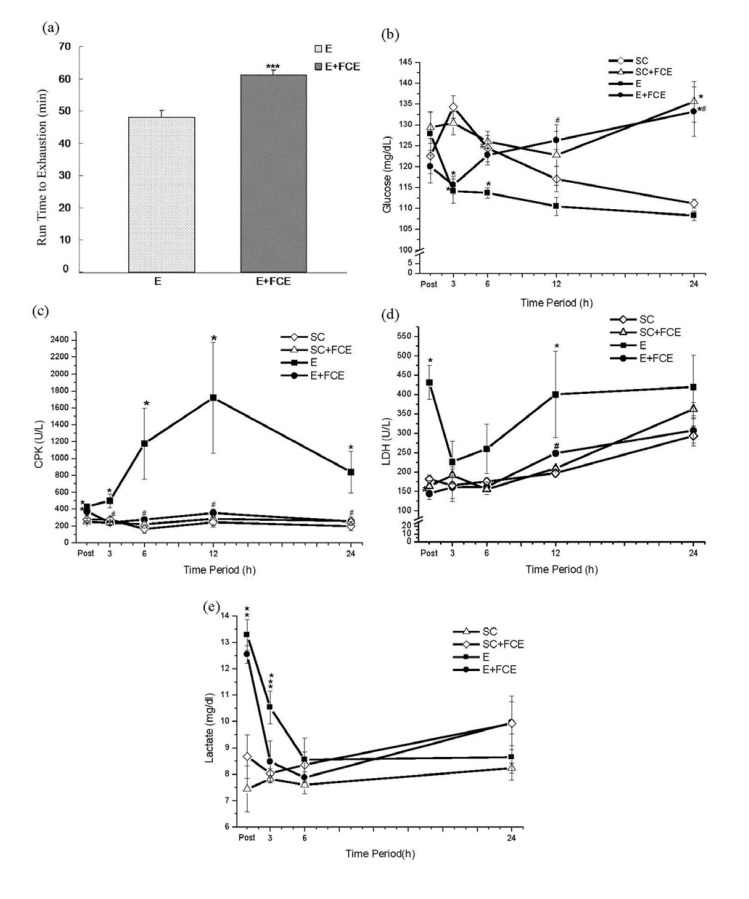
The run time to exhaustion on group E and E+FCE (**a**). Values are the means ± S.E.M.; n = 8. *** *p* < 0.001, compared with group E. Blood glucose (**b**), plasma CPK (**c**), plasma LDH (**d**) and plasma lactate (**e**) were obtained at several time points after exhaustive exercise. Values are the means ± S.E.M., * p < 0.05, compared with SC group; ^#^* p* < 0.05, compared with E group. The E+FCE rats were treated with FCE at a dose of 20 mg/kg body weight via orogastric gavage for 7 consecutive days before an acute bout of exhaustive exercise.

The mean run times to exhaustion for the E+FCE and E groups were 61.13 ± 1.47 and 48 ± 2.16 min, respectively. This showed a 27.4% higher endurance time for the E+FCE group in comparison with the E group (*p* < 0.001, Figure 1a).

### 2.3. Biochemical Parameters

The results showed that the plasma glucose levels of rats in the E group were lower than in the SC group. In such a system, FCE administration could rescue the exercise-induced reduction of glucose in the blood at 3 h after running (*p* < 0.05, Figure 1b). Likewise, FCE administration also stabilized the plasma glucose levels of rats without exercise (*p* < 0.05, Figure 1b). *Post hoc* analysis demonstrated that the lactate levels significantly increased at most time points after acute exercise in E group and E+FCE group when compared with the SC group and SC+FCE group (*p* < 0.05, Figure 1e), but there were no significant differences between the E group and E+FCE group (*p* > 0.05). Interestingly, the lactate level was still higher at 3 h in E group by comparison with other groups (*p* < 0.05, Figure 1e).

The plasma CPK and LDH (indicators of muscle damage) were followed. As Figure 1c,d show, the plasma LDH and CPK levels in the E group were significantly elevated after exhaustive exercise when compared to SC group and SC+FCE group (*p* < 0.05). Nonetheless, the plasma levels of CPK and LDH in the E+FCE group were significantly lower than the E group (*p* < 0.05, Figure 1c,d); the results were not significantly different compared to the SC or SC+FCE groups.

### 2.4. Pro-Inflammatory and Anti-Inflammatory Cytokines

**Figure 2:**
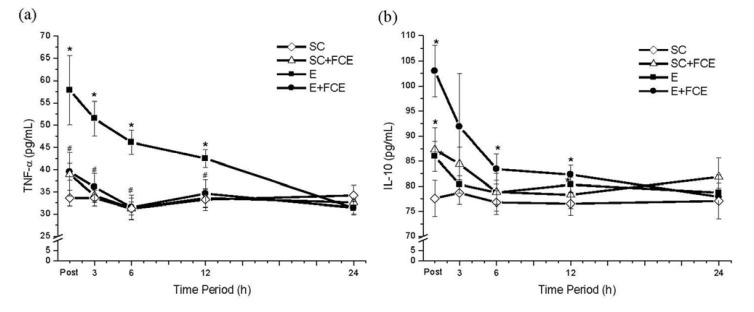
Pro-inflammatory cytokine TNF-α (**a**) and anti-inflammatory cytokine IL-10 (**b**) levels in plasma from groups SC, SC+FCE, E and E+FCE for varying times after exhaustive exercise. Values are the means ± S.E.M.; n = 8.  * p < 0.05, compared with SC group; ^#^* p* < 0.05, compared with E group.

As shown in Figure 2a, an acute bout of exhaustive exercise remarkably enhanced pro-inflammatory TNF-α cytokine in the E group. However, we observed that TNF-α level did not increase after running if the rats were treated with FCE (SC+FCE group). Additionally, FCE supplementation significantly increased the expression of anti-inflammatory IL-10 in the sera of rats in the E+FCE group and the SC+FCE groups compared to the E group or the SC groups (*p* < 0.05, Figure 2b).

### 2.5. Liver Function and Histological Evaluation

**Figure 3:**
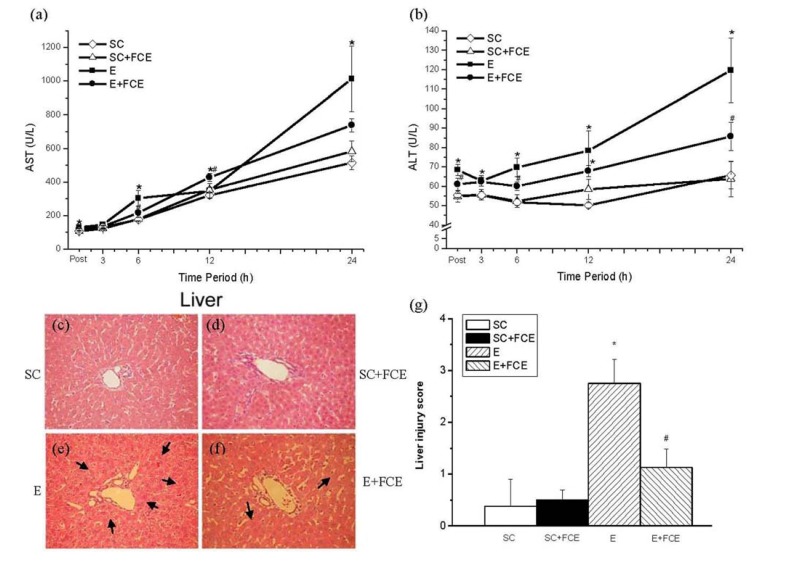
Assessment of the liver injury induced by intense exercise in rats. Measurement of plasma AST (**a**) and ALT (**b**) levels in experimental rats at post, 3, 6, 12, and 24 h after exercise. Values are the means ± S.E.M.; *n* = 8, * p < 0.05, compared to the SC group; ^#^* p* < 0.05, compared to the E group. Histologic sections from the liver (**c**–**f**) were stained with hematoxylin and eosin (H & E) to observe the tissue morphology infiltration (liver: original magnification 200×). Histopathologic injury score in the liver (**g**) after an acute bout of exhaustive exercise at 24 h in rats.* p < 0.05.

According to the ANOVA results the levels of plasma AST and ALT (biomarkers of liver function) were significantly elevated after exhaustive exercise in the E group, with peak values occurring at post and 24 h after running (*p* < 0.05, Figure 3a,b). FCE supplementation attenuated this raise, especially in the ALT levels. The plasma level of ALT in the E+FCE group was significantly lower than the E group at post, 6, 12, and 24 h post-exercise (*p* < 0.05, Figure 3b). After monitoring for 24 h, all of the rats were sacrificed for histological evaluation of H & E stained tissue sections from the liver. H & E stain of liver injury was confirmed by an increase of hemorrhage, cytoplasmic vacuolation, in the number of macrophages present (Figure 3c–f) and by the injury score (Figure 3g). As shown as in Figure 3e (*arrows*), an acute exhaustive exercise provoked more cytoplasmic vacuolation and serious macrophages infiltration compared to the other groups. Meanwhile, the data indicated that rats pre-administration with FCE had less macrophages infiltration in the H&E stain of liver (^#^ *p* < 0.05; Figure 3f,g) (*arrows*).

### 2.6. Discussion

The primary finding of this study was that FCE supplementation improved the endurance time to exhaustion and reduced muscle damage induced by an acute bout of exhaustive exercise. Besides, exhaustive exercise-induced inflammatory response and liver injury were also effectively attenuated by FCE administration.

In our study, we used the dosage of FCE based on a 60 kg human adult taking the commercially recommended dose of 1,200 mg of an FCE supplement daily [5]. We demonstrated that supplementation with FCE can prolong the run time to exhaustion in the exercised rats. It is known that the carbohydrate-protein (CHO-PRO) ingestion improved endurance exercise performance due to an increased rate of muscle glycogen storage during recovery [25]. Similar study also reported that a CHO-PRO beverage produced significant improvement in time to fatigue and diminutions in muscle damage in endurance athletes [26]. McCleave *et al*. [27] addressed that improvement in endurance performance occurred despite CHO-PRO supplement containing a lower CHO and caloric content in female athletes. Based on the data provided by Table 2, the major components of FCE (per 100 g) were 53.4 g protein and 30.4 g carbohydrates, respectively. Thus, we suggested that the endurance performance was affected by the supplementation of the protein and carbohydrate in FCE component. Meanwhile, the plasma glucose seemed to reflect the fatigue response after exhaustive exercise. Glucose concentrations, because of an equal rate of influx and efflux, at stages of rest are generally maintained at a state of equilibrium in the plasma. Intense exercise results in a glucose imbalance, with a higher rate of efflux from the plasma. Decreased plasma glucose levels parallel the onset of fatigue. The rate of glucose increase in the plasma is dictated by the amount of glucose being absorbed at the gut as well as hepatic glucose output. The ability of the liver to release glucose into the blood from glycogenolysis is unique because skeletal muscle, the other major glycogen reservoir, is incapable of doing so. Although gluconeogenesis is important in the maintenance of hepatic glucose stores, these stores alone cannot sustain exercise. For this reason, when glycogen stores are depleted during exercise, glucose levels fall and fatigue sets in. In the present study, with the administration of FCE, the decrease in plasma glucose caused by the exhaustive exercise protocol was significantly reduced, indicating that FCE can protect against exhaustive exercise-induced physiological stress. Correspondingly, plasma lactate accumulated immediately after intense exercise. Plasma lactate levels increased the concentration of hydrogen ions and decreased the pH within muscle or plasma [28]. An augmented lactate levels, although not causative, coincides with cellular acidosis and is a good indirect marker for the onset of fatigue [29]. In this study, there were significantly higher levels of plasma lactate in the E and E+FCE groups compared to SC and SC+FCE groups after exercise (post). Nonetheless, the E+FCE group generated lower levels of blood lactate at 3 h after intense exercise compared to the E group (Figure 1e). These observations proposed that FCE treatment served to delay acidosis and the time to exhaustion after exercise. Consistent with the above findings, the increase in the endurance time in the E+FCE group demonstrated that FCE might be beneficial in promoting improved endurance through stabilizing blood glucose levels and reducing the plasma lactate concentration.

**Table 2:** Approximate Composition of Freshwater Clam Extract (FCE) per 100 g.

Compoent	Amount
Protein (g)	53.4
Carbohydrates (g)	30.4
Crude fat (g)	11.0
Moisture (g)	1.1
Ash (g)	4.1

The levels of plasma LDH and CPK, two well-known biomarkers of tissue damage, were increased after exhaustive exercise. These observations support the belief that exercise-induced stress could significantly increase the risk of tissue damage [30]. An attractive result of this study was that FCE supplementation tended to diminish plasma LDH and CPK concentrations. It is generally believed that CHO-PRO or protein-based supplements affect hematological indicators and perceptions of pain after endurance exercise [26,31,32]. FCE is full of the relatively high protein content and the quantity of proteins in FCE could be important factors for protein metabolism and soreness following intense exercise. Skillen *et al*. [33] observed that the addition of amino acid to a carbohydrate beverage reduced muscle damage, decreased fatigue, and supported cycling performance. Furthermore, Etheridge *et al*. [34] found that when amino acid availability is increased via exogenous protein administration, the net protein balance increases to a net protein gain during the post-exercise period. Such an increase in protein synthesis could be expected to lead to a greater development of contractile proteins and increased restoration and enhancement of muscle contractile force.

Another important finding in this study was that the levels of the pro-inflammatory cytokine (TNF-α) and the anti-inflammatory cytokine (IL-10) were increased and decreased, respectively, in the E+FCE rats following heavy exertion. During inflammation, neutrophil mobilization and indiscriminate destruction of tissues by pro-inflammatory cytokines, such as TNF-α, may play a significant role in the pathogenesis of tissue injury. Therefore, TNF-α production may act as an important biomarker for the inflammatory response. Its level of expression could reflect the degree of inflammation in the body and provide a measurement of the effect of the medication on the inflammatory process [35]. In our experiment, there was a 148% increase in the pro-inflammatory TNF-α cytokine in the E group compared to the SC group; however, FCE administration effectively diminished the increased TNF-α expression in the E+FCE group. Besides, FCE treatment also caused a significant increase in IL-10. IL-10 is an anti-inflammatory cytokine that likely plays an important role in counteracting the inflammatory response due to exhaustive exercise.

FCE also contain s several dominant free amino acids, such as arginine, glutamic acid, glutamine, and alanine [36]. Arginine-enrichment could be an important factor for the anti-inflammatory effect of FCE. Numerous studies have shown that L-arginine is a precursor of NO, an important free radical scavenger, an inhibitor of neutrophil adhesion to the endothelium and a suppressor of the inflammatory response after exhaustive exercise [37]. Peng *et al*. [4] and Lee *et al*. [5] showed that FCE directly, through suppression of TNF-α and elevation of IL-10 in rats, ameliorated liver injury caused by hemorrhage. In our study, FCE was further illustrated to have a potent anti-inflammatory effect after exhaustive exercise.

In liver, glycogen can be metabolized and released to stabilize the glucose level in blood, which is associated with endurance performance. Likewise, the elevations of cytosolic enzymes in plasma, such as AST and ALT, are characteristic responses to intense exercise and are often used as indicators of liver injury [10]. In this study, both plasma ALT and AST levels were significantly elevated after an acute bout of exhaustive exercise in the E group, with peak values occurring at post and 24 h. FCE pretreatment attenuated this effect and diminished the increased levels of ALT. ALT is primarily found in the liver and more closely reflects liver damage or inflammation. An exhaustive exercise induces inflammation *in vivo* and damages multiple organs, such as the muscle [38], brain [39], lung [40], and heart [41] in rats. Only a few studies have been conducted to date that investigated the acute response of the liver to one single bout of exercise. It was reported that intense exercise caused histopathology changes [10] and alterations in the activities of antioxidant enzymes, lipid peroxidation, protein oxidation as well as the activity of 8-oxoG-DNA glycosylase in the liver of rodents [42,43]. The results from our study showed that the macrophages were recruited in the H & E staining of the liver (Figure 3e) after heavy exertion, but the score of peri-central vein macrophages infiltration in the liver could be decreased in the FCE treated group when compared to the E group (Figure 3g). Moreover, increased oxidative stress might be a major reason for the subsequent cellular stress in the liver. An acute bout of exhaustive exercise not only increases free radical production but also impairs the free-radical scavenging system, which exacerbates the liver’s ability to buffer large amounts of free radicals. Interestingly, FCE, by reducing reactive oxygen species (ROS) production in the hepatic tissue, could significantly reduce malondialdehyde (MDA) levels in a CCl_4_-induced liver damage [2]. The results from current study indicated that FCE attenuated both liver injury and inflammation induced by exhaustive exercise. For the above reasons, we propose that FCE has an important hepatoprotective role after exercise-induced injury.

## 3. Experimental

### 3.1. Animals

Thirty-two male Wistar-Kyoto strain (WKY) rats weighing 280~300 grams were used in this study. The animals were obtained from the National Animal Center and housed in the university animal center with sufficient air, a controlle d temperature (22 ± 1 °C) and a 12-hour light/dark cycle. The animals were supplied with food (standard rat chow) and water *ad libitum*. The study was approved by the Animal Care and Ethics Committee of Tzu Chi University, and animals received humane care in compliance with the “Principles of Laboratory Animal Care” developed by the National Society for Medical Research.

### 3.2. Preparation of Freshwater Clam Extract (FCE)

The freshwater clam extract was provided by Mike Biological Technologies (Taipei, Taiwan). The crude extract from the fresh clam was prepared according to a previous report [5]. The crude extracted powder from FCE was stored at −20 °C until used for the experiment. The approximate composition of 100 g of the powdered FCE (analyzed by SGS Taiwan, Ltd.) was demonstrated in Table 2.

### 3.3. Experimental Design

Thirty-two male rats were randomly divided into four groups (*n* = 8 each group): sedentary control (SC) and sedentary control with FCE supplementation (SC+FCE) groups, as well as exhaustive exercise (E) and exhaustive exercise with FCE supplementation (E+FCE) groups. The animals from the SC+FCE and E+FCE groups were treated with gavage administration of 20 mg/kg for seven consecutive days; SC and E groups were also given a vehicle for seven consecutive days prior to the exhaustive exercise test.

### 3.4. Exercise Protocol and Acute Experiment

All animals in the E and E+FCE groups were introduced to the motor-driven treadmill with 15~20-min exercise bouts at approximately 15~30 m/min for six days (each three days with the interval of 1-day) to familiarize them with the treadmill. The running protocol was modified based on the method by Lin *et al*. [44]. Electric shock was used to encourage the rats to run on the treadmill. On the day of the exercise experiment, the rats in E (*n* = 8) and E+FCE (*n* = 8) groups were required to run to exhaustion on a six-lane inclined (10°) treadmill at a final speed of 30 m/min, which was approximately 70–75% of VO_2_max [45]. Exhaustion was defined as the time when the rats could no longer keep pace with the treadmill speed [46]. To eliminate diurnal effects, the experiments were implemented at the same time of day (09:00 to 12:00).

After the acute exercise test, we used a conscious model developed in our laboratory [4,5,47,48,49]. Rats were anesthetized with ether inhalation for approximately 15 min in the surgical procedure. While under anesthesia, a polyethylene catheter (PE-50) was inserted into the right femoral artery to collect blood samples. The operation was completed within 10 min, and the incision was less than 0.5 cm^2^. All procedures were performed under sterile conditions. After the operation, the animals were placed in a metabolic cage (Shingshieying Instruments, Hualien, Taiwan) and were awakened soon thereafter. The rats were allowed free access to food and water. With this method, the rats could be continuously monitored for 24 h; blood was collected at different time points while the rats were conscious.

### 3.5. Blood Collection and Determination of Biochemical Parameters

Blood samples (0.5 mL) were obtained from the femoral arterial at 0.5 (post), 3, 6, 12, and 24 h after exhaustive exercise test, and an equal volume of 0.5 mL normal saline was used for fluid resuscitation. The blood samples were immediately placed into heparinized tubes and centrifuged at 3,000 *g* for 10 min. The plasma was decanted and separated into two parts; one part was stored at 4 °C within 1 h after collection for biochemical analysis. Plasma levels of blood glucose, lactate, creatine phosphokinase (CPK), lactate dehydrogenase (LDH), aspartate aminotransferase (AST), and alanine aminotransferase (ALT) were measured with an autoanalyzer (COBAS Integra C111; Roche Diagnostics, Basel, Switzerland). The other part of the plasma was stored at −20 °C for later measurements of TNF-α and IL-10 concentrations.

### 3.6. Measurement of TNF-α and IL-10

TNF-α and IL-10 concentrations in the plasma were measured using antibody enzyme-linked immunosorbent assays (ELISAs) with commercial antibody pairs, recombinant standards, and a biotin streptavidin-horseradish peroxidase detection system (DuoSet, R&D systems, Minneapolis, MN, USA). The ELISA was performed following the manufacturer’s instructions and analyzed by optical density using a 96-well microplate reader (Bio-Rad, Hercules, CA, USA) at 450/540 nm wavelength. All measurements were executed in duplicate. The coefficients of variation (CV) were less than 10% for IL-10 and 20% for TNF-α.

### 3.7. Histological Examination of the Liver

The liver was removed immediately after sacrifice at 24 h post-exhaustive exercise. The liver specimens were fixed overnight in 4% buffered formaldehyde, processed using standard methods, and stained for hematoxylin and eosin (H&E). The stained organ specimens were analyzed by a blinded observer. The severity of liver injury observed in the tissue sections was scored as follows: 0, no evidence or minimal evidence of injury; 1, mild injury consisting of cytoplasmic vacuolation and focal nuclear pyknosis; 2, moderate to severe injury with extensive nuclear pyknosis, cytoplasmic hypereosinophilia, and loss of intercellular borders; and 3, severe necrosis with disintegration of the hepatic cords, haemorrhage, and neutrophil infiltration [4,50]. All assessments were made on five fields per section and five sections per liver.

### 3.8. Statistical Analysis

All data were expressed as the mean ± S.E.M. The significance of differences among values was analyzed by one-way ANOVA followed by Duncan’s test. Histological scores were analyzed using the Kruskal-Wallis test. Differences were considered to be statistically significant at a *p* value < 0.05 .The Statistical Software Package for the Social Sciences, version 18.0 for Windows (SPSS Inc., Chicago, IL, USA) was used.

## 4. Conclusions

In conclusion, this investigation has successfully demonstrated that FCE administration could extend the run time to exhaustion and reduce exercise-induced muscle damage along with inflammation in rats. Thus, we kindly suggest that FCE might provide the beneficial effects required for improving endurance performance if it is used as a daily ergogenic aid during the training period. However, the bioactive components in freshwater clams extract that conduced to detailed anti-fatigue mechanisms and hepatoprotective effects are necessary to be further clarified.
